# Sympathetic Neurotransmitters Modulate Osteoclastogenesis and Osteoclast Activity in the Context of Collagen-Induced Arthritis

**DOI:** 10.1371/journal.pone.0139726

**Published:** 2015-10-02

**Authors:** Dominique Muschter, Nicole Schäfer, Hubert Stangl, Rainer H. Straub, Susanne Grässel

**Affiliations:** 1 Department of Orthopedic Surgery, Experimental Orthopedics, University Hospital Regensburg, Regensburg, Bavaria, Germany; 2 Department of Internal Medicine I, Laboratory of Experimental Rheumatology and Neuroendocrine Immunology, University Hospital Regensburg, Regensburg, Bavaria, Germany; 3 Center for Medical Biotechnology, BioPark I, Regensburg, Bavaria, Germany; Faculté de médecine de Nantes, FRANCE

## Abstract

Excessive synovial osteoclastogenesis is a hallmark of rheumatoid arthritis (RA). Concomitantly, local synovial changes comprise neuronal components of the peripheral sympathetic nervous system. Here, we wanted to analyze if collagen-induced arthritis (CIA) alters bone marrow-derived macrophage (BMM) osteoclastogenesis and osteoclast activity, and how sympathetic neurotransmitters participate in this process. Therefore, BMMs from Dark Agouti rats at different CIA stages were differentiated into osteoclasts *in vitro* and osteoclast number, cathepsin K activity, matrix resorption and apoptosis were analyzed in the presence of acetylcholine (ACh), noradrenaline (NA) vasoactive intestinal peptide (VIP) and assay-dependent, adenylyl cyclase activator NKH477. We observed modulation of neurotransmitter receptor mRNA expression in CIA osteoclasts without affecting protein level. CIA stage-dependently altered marker gene expression associated with osteoclast differentiation and activity without affecting osteoclast number or activity. Neurotransmitter stimulation modulated osteoclast differentiation, apoptosis and activity. VIP, NA and adenylyl cyclase activator NKH477 inhibited cathepsin K activity and osteoclastogenesis (NKH477, 10^-6^M NA) whereas ACh mostly acted pro-osteoclastogenic. We conclude that CIA alone does not affect metabolism of *in vitro* generated osteoclasts whereas stimulation with NA, VIP plus specific activation of adenylyl cyclase induced anti-resorptive effects probably mediated via cAMP signaling. Contrary, we suggest pro-osteoclastogenic and pro-resorptive properties of ACh mediated via muscarinic receptors.

## Introduction

One of the most severe characteristics of rheumatoid arthritis (RA) is the destruction of diarthrodial joint bony tissue leading to disability and disuse. Main mediator cells are osteoclasts, a unique cell type able to degrade rigid bone matrix [1]. Osteoclasts are derived from the monocyte-macrophage lineage of the hematopoietic stem cell population residing within the bone marrow [2]. The differentiation of osteoclasts is mainly dependent on two essential factors: macrophage colony-stimulating factor (M-CSF) and receptor activator of NFκB ligand (RankL), the first being indispensable for proliferation and survival of macrophages [3, 4] and the latter being the key inducer of osteoclast formation [5].

Neurotransmitters released from nerve endings or resident cells provide additional modulatory potential for osteoclast development and activity. *In vivo* studies showed that catecholaminergic noradrenaline (NA) and cholinergic acetylcholine (ACh) / peptidergic vasoactive intestinal peptide (VIP) affect bone homeostasis oppositely. NA signaling preferentially leads to a reduced bone mass phenotype *in vivo*, either by β2- or by α-adrenoceptor (AR) mediated responses [6–8]. *In vitro-*studies, using murine or human osteoclast-like cells, showed expression of α- and β-adrenoceptor mRNA and enhanced osteoclastogenesis specifically upon β-adrenoceptor agonism [9, 10]. Oppositely, *in vivo*-studies, using VIP self-associated in micelles for nano-medicine application, as well as nicotine and nicotinic α7 ACh receptor agonist ARR–17779, prevented arthritis-induced bone loss [11, 12]. *In vitro*, VIP inhibits osteoclast resorption and cholinergic agonist carbamylcholine induced osteoclast apoptosis [13, 14]. Classical VIP receptor and β2-adrenoceptor signaling is associated with enhanced cyclic AMP levels [15, 16] known to modulate inflammation and initiate predominantly anti-inflammatory mechanisms [17].

Alterations associated with inflammatory arthritis affect the local joint innervation and the respective neurotransmitter microenvironment. Tyrosine hydroxylase (TH)-positive nerve fibers, the marker for catecholaminergic nerves, disappear in synovial membrane regions and are instead replaced by peripheral acting TH-positive cells providing a local source for NA [18, 19]. The number of VIP-immunoreactive fibers was unaltered in inflamed ankle joints of arthritic rats but VIP concentration was enhanced [20]. Little is known about local synovial cholinergic innervation in inflammatory arthritis so far, but a non-neuronal cholinergic system, consisting of fibroblast-like and mononuclear-like cells, with enhanced outflow in arthritis has been reported by Grimsholm et al. [21]. Notably, our group recently reported that in less inflamed, not arthritis affected joint adjacent tissues as muscle and skin but not in inflamed synovial tissues, a catecholaminergic-to-cholinergic transition occurs [22].

Here, we ask whether the observed alterations in neurotransmitter supply will have an impact on osteoclast development and function *in vitro* during collagen-induced arthritis (CIA) progression. For the purpose of this study we used a CIA model in Dark Agouti (DA) rats, where we isolated bone marrow-derived macrophages (BMMs) from arthritic rats in different disease stages and from age-matched sodium chloride (NaCl)-treated controls. M-CSF/RankL-induced osteoclastogenesis and osteoclast activity was studied *in vitro* in the presence of neurotransmitters NA, ACh and VIP. The results of this study provide novel information how catecholaminergic and cholinergic / peptidergic neurotransmitters alter osteoclast development and function and that progression of CIA has only little influence on osteoclast metabolism.

## Results

### 1. Influence of collagen-induced arthritis on neurotransmitter receptor gene and protein expression

**Fig 1:**
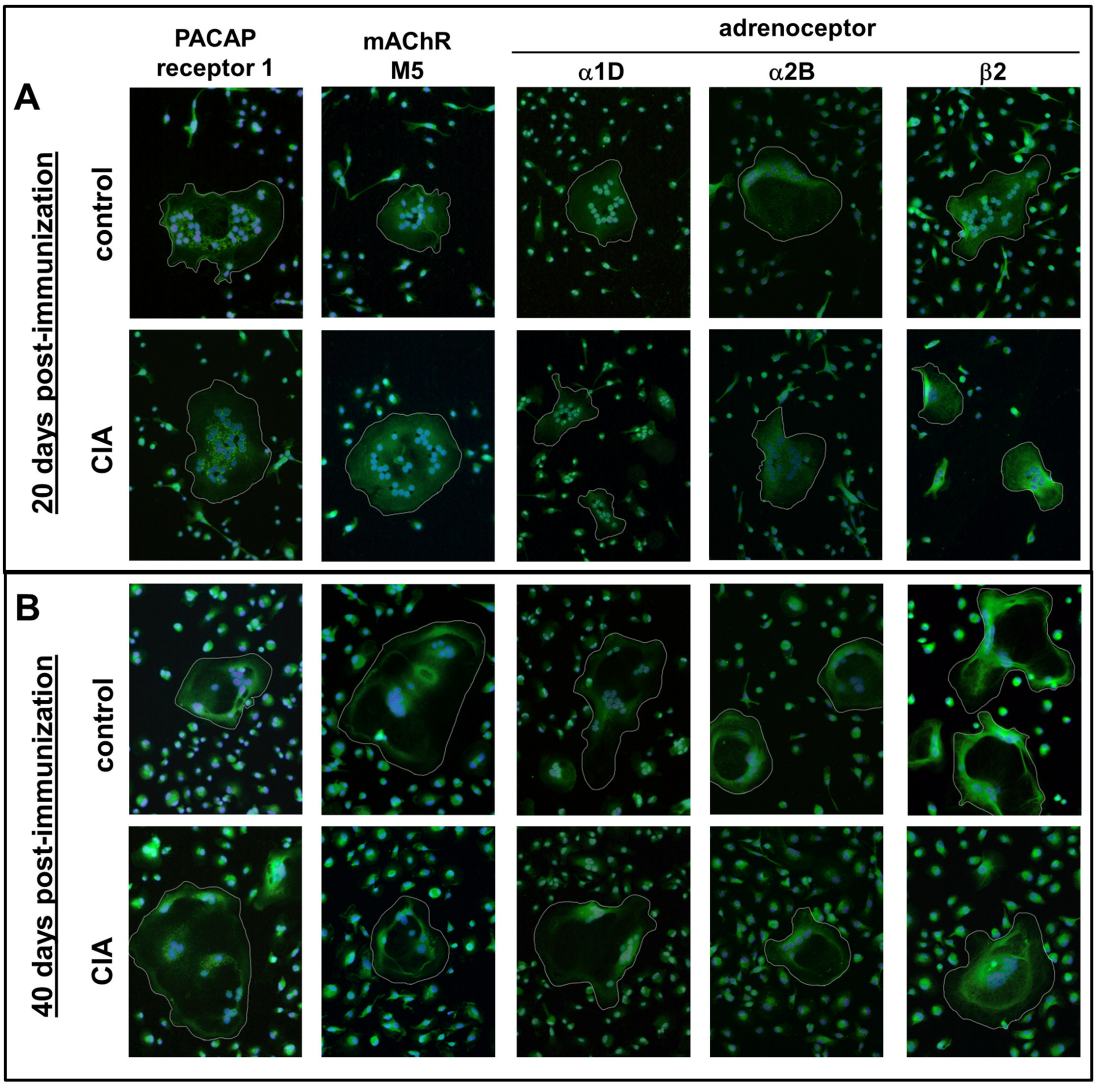
Analysis of neurotransmitter receptors by immunofluorescence staining. Adrenergic receptors α1D, α2B, β2; muscarinic ACh receptor M5 and the alternative VIP receptor PACAP receptor 1 were stained positive on osteoclasts derived from BMMs of controls and CIA animals 20 (A) and 40 (B) days post-immunization. Paraformaldehyde-fixed cells were stained after 5 days differentiation. Nuclei were counterstained with DAPI. Cells containing 3 or more nuclei are considered to be osteoclasts (white circles). N = 4 rats. Magnification 200x. ACh: acetylcholine, AR: adrenoceptor, BMM: bone marrow-derived macrophages, CIA: collagen-induced arthritis, mAChR: muscarinic ACh receptor, NA: noradrenaline, PACAP: pituitary adenylate cyclase-activating peptide, VIP: vasoactive intestinal peptide

First, we verified the expression of receptors for ACh, NA and VIP by osteoclasts on mRNA and protein level (Table 1, Fig 1). CIA constantly suppressed VIP receptor 1 mRNA expression at all time-points (day 10: asymptomatic phase, day 15: disease onset, day 20: acute inflammatory phase, day 40: chronic phase) in relation to osteoclasts from controls (Table 1 part A). VIP receptor 2 mRNA was down-regulated 10 days post-immunization (p.i.), whereas PACAP receptor 1 mRNA was downregulated from day 15 p.i. until day 40 p.i (Table 1 part A). Additionally, adrenoceptor β2 was downregulated by CIA at all time points. Fewer effects were seen for adrenoceptors α1D and α2B, which were downregulated at days 20 and 10 p.i., respectively (Table 1 part A). CIA effects on muscarinic ACh receptors M3 and M5 expression were dependent on the respective arthritis-stage. In stages with little inflammation, like 10 and 40 days p.i., both were downregulated in osteoclasts from CIA animals. Instead, 20 days p.i. associated with high-grade joint inflammation, mRNA expression of ACh receptors M3 and M5 was significantly upregulated by CIA. Particularly, the M5 ACh receptor mRNA was strongly affected by CIA (Table 1 part A). In contrast to gene expression data, protein expression and cellular location of receptors showed no obvious differences when assessed by qualitative immunofluorescence staining (Fig 1).

**Table 1:** Relative quantitative real-time PCR.

	10 days p. I.	15 days p. I.	20 days p. I.	40 days p. I.
**A) Neurotransmitter receptor genes**				
VIP receptor1 (n = 13–15)	***-1,13±0,17	***-0,91±0,13	***-0,53±0,09	***-0,56±0,12
VIP receptor2 (n = 13–15)	**-0,86±0,26	-0,11±0,17	0,31±0,31	-0,27±0,16
PACAP receptor1 (n = 8–14)	-0,95±0,58	*-0,80±0,29	**-0,46±0,14	**-0,72±0,24
AR α1D (n = 5–10)	-1,92±0,72	-0,06±0,78	*0,96±0,37	-0,83±0,59
AR α2A (n = 10–13)	-0,54±0,41	-0,39±0,32	-0,20±0,28	0,02±0,16
AR α2B (n = 5–11)	*-1,42±0,48	0,25±0,72	0,92±0,28	-0,94±0,42
AR β2 (n = 10–13)	**-1,07±0,15	**-0,86±0,19	**-0,36±0,27	*-0,31±0,11
mAChR M3 (n = 10–15)	*-0,70±0,33	-0,60±0,37	*0,59±0,26	**-0,92±0,28
mAChR M5 (n = 5–13)	*-3,51±0,73	0,68±0,83	**2,75±0,85	*-1,41±0,58
**B**) **Osteoclast differentiation marker genes**				
M-CSF receptor (n = 7–12)	**-0,66±0,16	**-0,30±0,10	-0,28±0,17	-0,17±0,14
Rank receptor (n = 10–15)	**-0,63±0,11	**-0,60±0,19	-0,05±0,31	*-0,30±0,14
NFATc1 (n = 10–11)	*-0,39±0,17	**-0,46±0,12	-0,62±0,31	**-0,35±0,10
Calcitonin receptor (n = 12–15)	***-1,19±0,25	**-0,65±0,21	-0,43±0,33	-0,28±0,21
**C) Survival / apoptosis-related genes**				
Erk1 (n = 8–13)	**-0,60±0,12	**-0,28±0,08	*-0,25±0,10	-0,03±0,11
Erk2 (n = 8–12)	**-0,54±0,15	*-0,21±0,08	-0,21±0,11	-0,09±0,11
Akt1 (n = 7–12)	*-0,44±0,19	-0,16±0,09	*-0,25±0,14	-0,03±0,11
c-myc (n = 8–12)	0,12±0,12	***0,69±0,15	-0,02±0,12	*0,71±0,32
Bcl–2 (n = 11)	*-0,44±0,17	***-0,69±0,11	**-0,87±0,26	*-0,18±0,08
**D) Osteoclast activity marker genes**				
Cathepsin K (n = 12–16)	*-0,52±0,21	**-1,09±0,29	-0,50±0,23	-0,11±0,15
MMP9 (n = 12–15)	***-0,64±0,10	*-0,60±0,21	-0,20±0,20	-0,21±0,14
TRAP (n = 10–12)	-0,24±0,40	*-0,77±0,27	**-0,81±0,18	-0,19±0,16
Carbonic anhydrase II (n = 10–12)	**-0,54±0,14	*-0,51±0,21	-0,48±0,29	-0,02±0,16
Tcirg1 (n = 6–8)	-0,06±0,18	-0,22±0,35	-0,52±0,24	*-0,25±0,06

Table 1 summarizes gene expression data of osteoclasts generated from BMM isolated from arthritic rats 10, 15, 20 and 40 days following immunization after 5 days of osteoclastogenic differentiation. Gene expression data of osteoclasts from arthritic rats are presented relative to gene expression of time-matched osteoclasts derived from BMM isolated from control rats. Neurotransmitter receptor genes are presented under **A**, osteoclast differentiation marker genes under **B**, genes related to cell apoptosis and survival under **C** and osteoclast activity marker genes under **D**. Data are expressed as mean ± standard error of the mean of the log fold change to calibrator (base 2). Genes of interest were normalized to GAPDH expression.

*p<0,05

**p<0,01

***p<0,001. Numbers in brackets represent the number of rats analyzed.

As we confirmed the existence of various receptors for ACh, NA and VIP on *in vitro*-generated osteoclasts, we further applied these neurotransmitters in functional osteoclast assays to study their impact on osteoclastogenesis and activity under healthy and arthritic conditions.

### 2. Osteoclast numbers and gene expression analysis of differentiation markers

**Fig 2:**
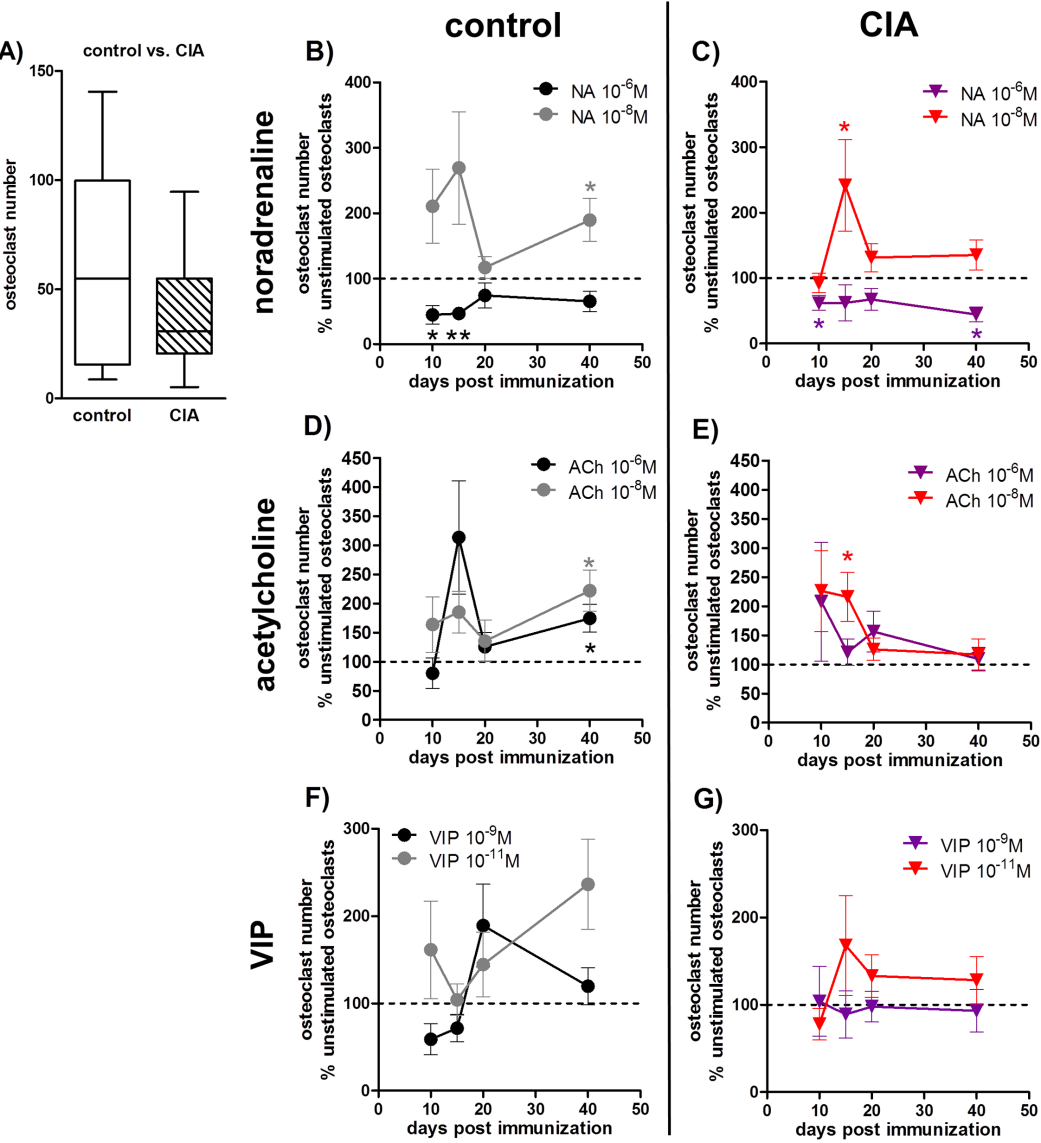
Analysis of osteoclast numbers. After 5 days of differentiation, TRAP-positive cells containing three or more nuclei were defined as osteoclasts. **A)** shows osteoclast numbers generated from BMMs of CIA rats 40 days p.i. and the respective osteoclast numbers from control rats. N = 15 rats. **B-G) The effect of neurotransmitter stimulation on osteoclast numbers from CIA and control rats is shown as percentage to respective unstimulated osteoclasts in the time-course of arthritis.** The results for NA stimulation are presented under **B** and **C** and the results for ACh and VIP stimulation are presented under **D, E** and **F, G**, respectively (unstimulated osteoclasts = 100%, dotted line). N (control/CIA rats) = NA 10^-6^M 10d (8/8), 15d (8/7), 20d (7/8), 40d (9/8); NA 10^-8^M 10d (8/7), 15d (8/7), 20d (7/8), 40d (9/8); ACh 10^-6^M 10d (7/7), 15d (9/9), 20d (7/8), 40d (9/8); ACh 10^-8^M 10d (8/8), 15d (9/9), 20d (7/8), 40d (9/8), VIP 10^-9^M 10d (8/8) 15d (9/9) 20d (7/8) 40d (8/8), VIP 10^-11^M 10d (8/7) 15d (9/9) 20d (7/8) 40d (9/8). Assay was performed in duplicate. Box plots represent the 10^th^ to 90^th^ percentile of data sets. Data points show mean ± SEM. *p<0,05, **p<0,01. ACh: acetylcholine, BMM: bone marrow-derived macrophage, CIA: collagen-induced arthritis, NA: noradrenaline, SEM: standard error of the mean, p.i.: post-immunization, TRAP: tartrate-resistant acid phosphatase

The influence of CIA and ACh, NA and VIP on osteoclastogenesis was analyzed by determining the number of osteoclasts formed after 5 days of differentiation from BMMs isolated from arthritic and control animals (Fig 2). CIA had no impact on osteoclast numbers in relation to osteoclast numbers from controls (Fig 2A). Notably, mRNA expression of major osteoclast differentiation marker genes like transcription factor NFATc1, calcitonin, Rank and M-CSF receptor, was significantly suppressed by CIA 10 and 15 days p.i., and for Rank receptor and NFATc1 additionally at day 40 p.i. (Table 1 part B).

#### Neurotransmitter stimulation effects on osteoclast formation are presented as percentage to the respective unstimulated cultures from CIA and control rats in the time-course of arthritis

NA effects were dose-dependent and the effect intensity changed over time (Fig 2B and 2C). 10^-6^M NA inhibited osteoclast formation significantly in osteoclasts derived from BMMs of CIA animals 10 and 40 days p.i. (Fig 2C) whereas osteoclastogenesis of control BMMs was significantly suppressed 10 and 15 days after treatment with NaCl (Fig 2B). Stimulatory effects of 10^-8^M NA were strongest in CIA osteoclasts 15 days p.i. (Fig 2C) and in control osteoclasts 40 days after NaCl treatment in relation to their respective unstimulated cultures (Fig 2B). 10^-6^M and 10^-8^M ACh profoundly stimulated osteoclastogenesis in controls relative to unstimulated cultures 40 days after NaCl treatment (Fig 2D), while CIA BMM osteoclastogenesis was stimulated by 10^-8^M ACh 15 days p.i. (Fig 2E). VIP had no statistically significant effects on osteoclast numbers (Fig 2F and 2G).

### 3. Analysis of caspase 3/7-mediated apoptosis and gene expression of apoptosis-related markers

**Fig 3:**
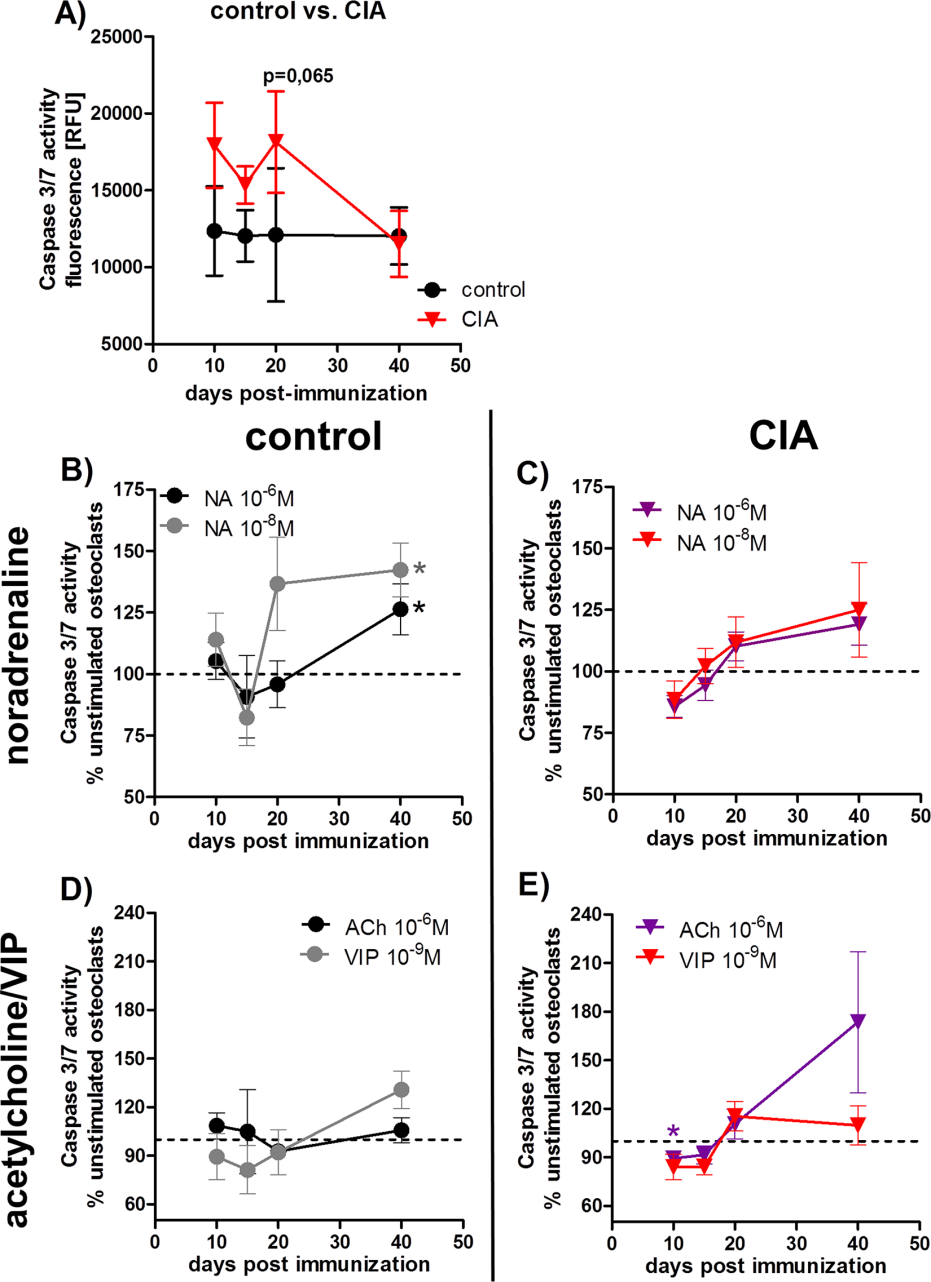
Analysis of caspase 3/7 activity in osteoclasts. 24 hours serum-deprived osteoclasts from control and CIA rats were incubated with caspase 3/7 reagent for 6–10 hours. Osteoclast caspase 3/7 activity of control and CIA osteoclasts in the time-course of arthritis is shown under **4A.** N (control/CIA rats): 10d (6), 15d (8), 20d (8), 40d (8). Influence of stimulation with NA (**B, C**) and ACh / VIP (**D, E**) on caspase 3/7 activity in osteoclasts from control and CIA rats is shown as **percentage to respective unstimulated osteoclasts** from CIA and control rats in the time-course of disease progression (unstimulated osteoclasts (100%) = dotted line). N (control/CIA rats) = NA 10^-6^M 10d (6/6), 15d (6/6), 20d (6/6), 40d (7/7); NA 10^-8^M 10d (6/6), 15d (6/6), 20d (6/6), 40d (8/7); ACh 10^-6^M 10d (6/6), 15d (6/6), 20d (5/6), 40d (7/8); VIP 10^-9^M 10d (6/6), 15d (6/6), 20d (5/6), 40d (8/7). Assay was performed in triplicate. Box plots represent the 10^th^ to 90^th^ percentile of data sets. Data points show mean ± SEM.*p<0,05. ACh: acetylcholine, CIA: collagen-induced arthritis, NA: noradrenaline, VIP: vasoactive intestinal peptide

To analyze the influence of CIA and neurotransmitter-mediated effects on apoptosis, we analyzed caspase 3/7 activity at different stages of CIA. We observed little changes in caspase 3/7-mediated apoptotic activity during CIA except for 20 days p.i., where osteoclasts derived from CIA BMMs were by trend more sensitive to apoptosis induction (Fig 3A).

#### Effects of neurotransmitter stimulation on caspase 3/7 activity are presented as percentage to the respective unstimulated cultures from CIA and control rats

Induction of apoptosis was observed upon stimulation with 10^−6^ and 10^-8^M NA in osteoclasts derived from BMMs of control animals compared with unstimulated cultures 40 days after NaCl treatment (Fig 3B). Contrary, 10^-6^M ACh inhibited caspase 3/7 activity in osteoclasts derived from BMMs of arthritic rats 10 days p.i. (Fig 3E) whereas VIP did not affect caspase 3/7 activity of osteoclasts (Fig 3D and 3E).

Furthermore, we analyzed gene expression of M-CSF receptor-related signaling molecules involved in pathways regulating cell survival and apoptosis e.g. kinases like survival-enhancing Akt1 [23], MAP kinases Erk1 and Erk2 which are involved in pro-apoptotic signaling pathways [24], the pro-apoptotic protein c-myc [25] and the anti-apoptotic protein Bcl–2 [26]. Latter was found to be constantly down-regulated in osteoclasts derived from BMMs of arthritic animals in the time-course of CIA (Table 1 part C). Erk1 mRNA was downregulated from day 10 until day 20 p.i., Erk2 mRNA at days 10 and 15 and Akt1 mRNA at days 10 and 20 p.i. in relation to mRNA of osteoclasts derived from control animals at the respective time-points (Table 1 part C). Oppositely, c-myc mRNA was significantly upregulated in osteoclasts differentiated from BMM of arthritic rats 15 and 40 days p.i. (Table 1 part C).

### 4. Evaluation of cathepsin K enzyme activity

**Fig 4:**
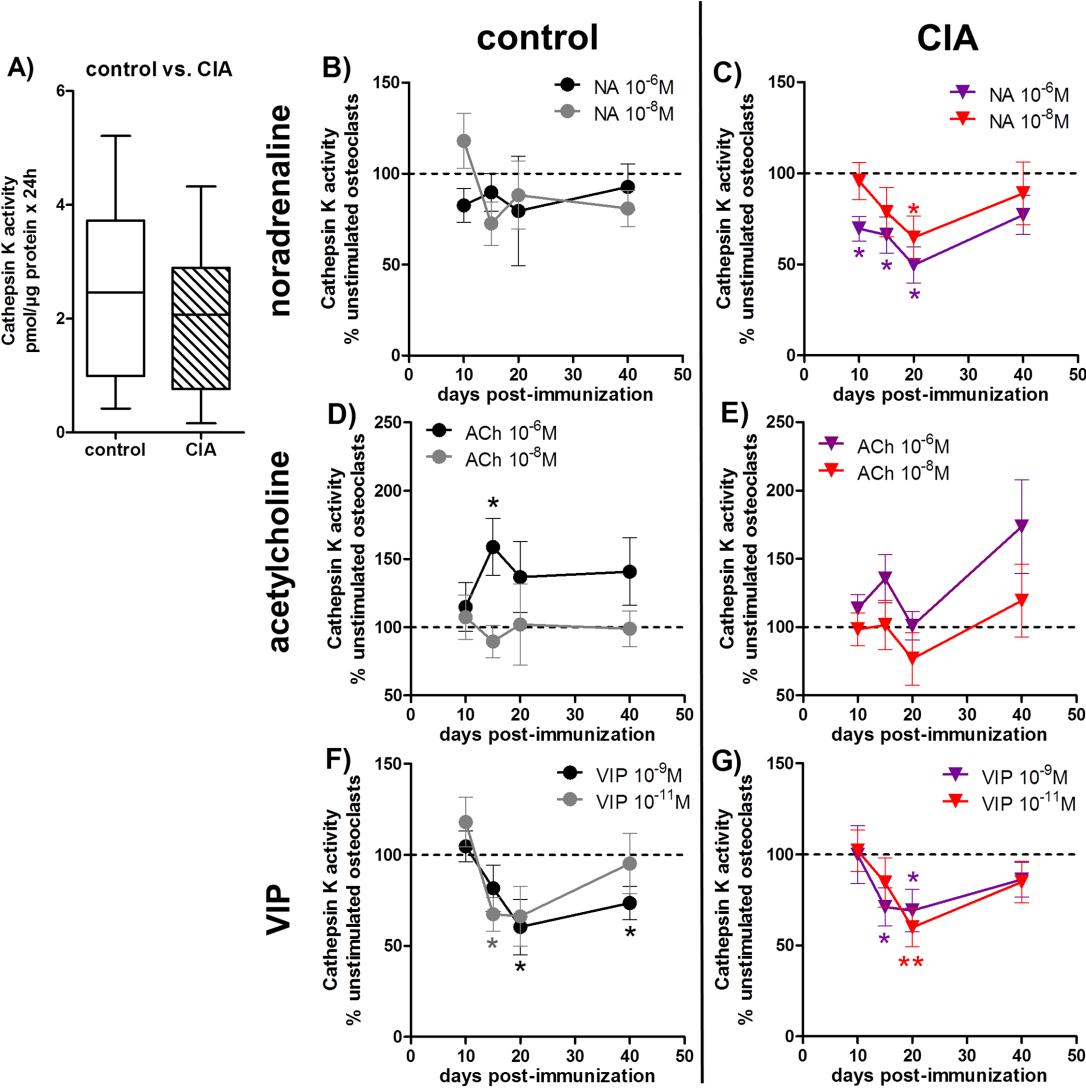
Evaluation of osteoclast cathepsin K enzyme activity. After 5 days of differentiation, osteoclasts were incubated in serum-free medium for another 24 hours and the collected supernatant was analyzed for cathepsin K enzyme activity. **A)** shows the cathepsin K activity of osteoclasts from CIA rats 40 days p.i. and the respective cathepsin K activity of osteoclasts from control rats. N = 14 rats. **B-G) The effect of neurotransmitter stimulation on cathepsin K enzymatic activity of osteoclasts from CIA and control rats is shown as percentage to respective unstimulated osteoclasts in the time course of arthritis.** The results for NA are presented under **B** and **C,** for ACh under **D, E** and for VIP stimulation under **F, G** (unstimulated (100%) = dotted line). N (control/CIA rats) = NA 10^-6^M 10d (10/11) 15d (14/14) 20d (12/13) 40d (14/12); NA 10^-8^M 10d (10/11) 15d (13/13) 20d (12/14) 40d (13/12); ACh 10^-6^M 10d (10/11) 15d (13/14) 20d (12/12) 40d (13/12); ACh 10^-8^M 10d (11/11) 15d (13/14) 20d (12/13) 40d (13/12); VIP 10^-9^M 10d (10/11) 15d (14/13) 20d (12/14) 40d (13/12); VIP 10^-11^M 10d (10/12) 15d (13/14) 20d (12/13) 40d (13/12). Cells were seeded in duplicate for osteoclastogenesis and each supernatant was analyzed in duplicate. Box plots represent the 10^th^ to 90^th^ percentile of data sets. Data points show mean ± SEM. *p<0,05, **p<0,01, ***p<0,001. ACh: acetylcholine, CIA: collagen-induced arthritis, NA: noradrenaline, p.i.: post-immunization, VIP: vasoactive intestinal peptide

As a standard marker for osteoclast activity, we studied cathepsin K activity in cell culture supernatants of *in vitro*-generated osteoclasts from BMMs of CIA and control rats (Fig 4).

Cathepsin K activity in the cell culture supernatants of osteoclasts generated from BMMs of arthritic animals in relation to osteoclast supernatants from controls was unaltered at all time points. Fig 4A shows representative results for day 40 p.i., whereas cathepsin K mRNA expression was downregulated in CIA-derived osteoclasts 10 and 15 days p.i. (Table 1 part D).

#### Neurotransmitter stimulation effects on cathepsin K activity are presented as percentage to the respective unstimulated cultures from CIA and control rats in the time-course of arthritis

We observed catecholaminergic inhibition of cathepsin K activity with 10^-6^M NA in culture supernatants of CIA-derived osteoclasts from day 10 until day 20 p.i. and for 10^-8^M NA at day 20 relative to unstimulated cultures (Fig 4C). NA effects on cathepsin K activity of controls did not reach significance with regard to specific time points (Fig 4B). In contrast to NA, 10^-6^M ACh enhanced cathepsin K activity in the cell culture supernatants of control-derived osteoclasts 15 days post NaCl treatment (Fig 4D). Dissection of specific arthritis time-points highlights, that 10^-9^M VIP mainly inhibited CIA osteoclast cathepsin K activity 15 and 20 days p.i. (Fig 4G) and, slightly delayed, 20 and 40 days post NaCl-treatment (Fig 4F). 10^-11^M VIP affected osteoclasts differentiated from CIA BMMs 20 days p.i. (Fig 4G) and osteoclasts from controls 15 days past NaCl-treatment (Fig 4F).

### 5. Influence of adenylyl cyclase stimulation on osteoclast formation and cathepsin K activity

Because NA and VIP signal transduction is associated with increasing cAMP levels, we used the adenylyl cyclase activator NKH477 to specify if the reduced cathepsin K activity might be mediated by cAMP-associated pathways and how osteoclastogenesis would be affected in the presence of NKH477.

#### Effects of NKH477 on osteoclastogenesis and cathepsin K activity are presented as percentage to the respective unstimulated osteoclast cultures from CIA and control rats

**Fig 5:**
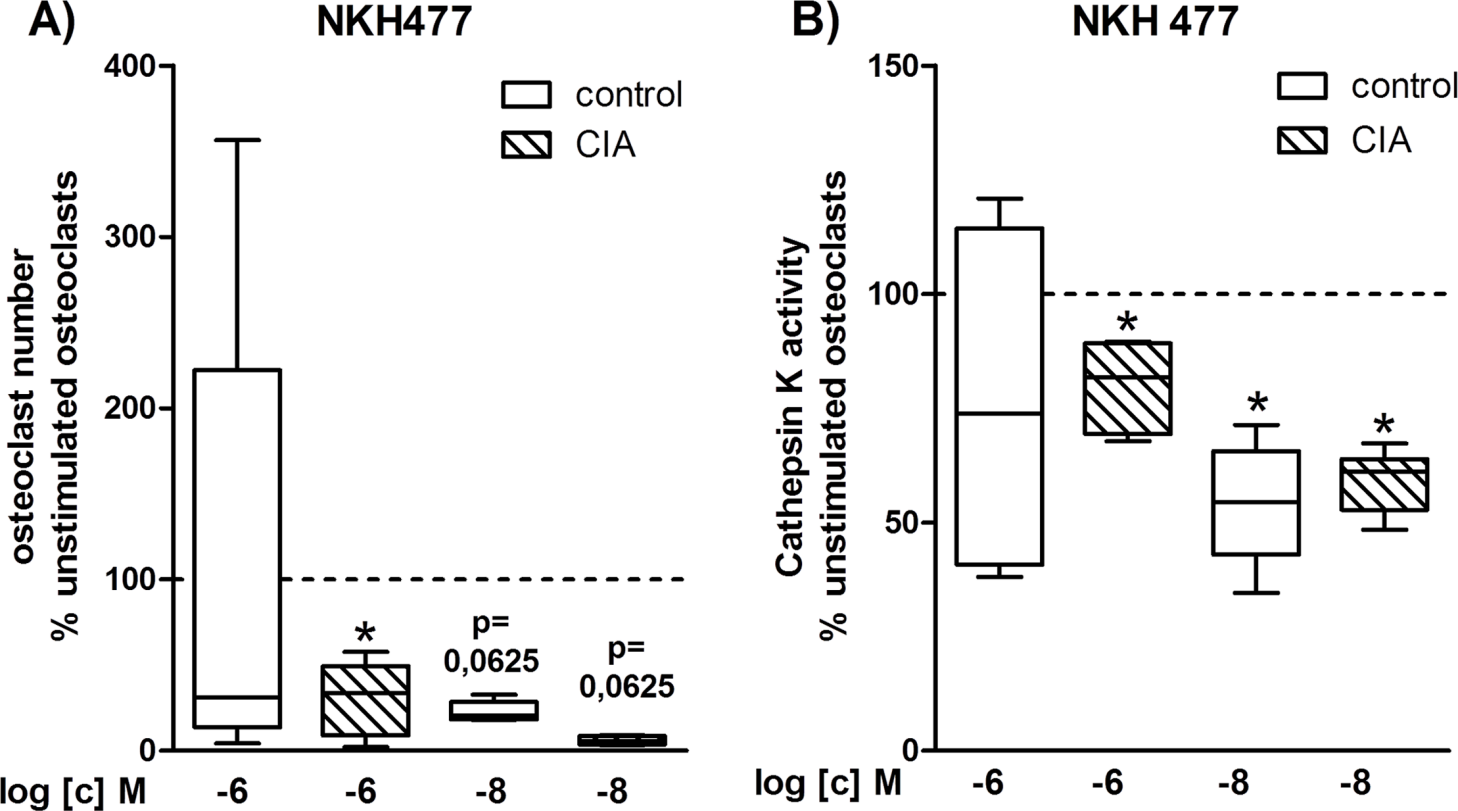
Influence of cAMP signaling on osteoclast number and cathepsin K activity. After 5 days of differentiation, osteoclasts were incubated in serum-free medium for another 24 hours and the collected supernatant was analyzed for cathepsin K enzyme activity. Remaining cells were fixed, stained for TRAP and cells containing ≥ 3 nuclei were counted as osteoclasts. The effect of adenylyl cyclase activator NKH 477 on osteoclast number **(A)** and osteoclast cathepsin K enzymatic activity **(B)** from CIA rats 40 days p.i. and control rats 40 days post NaCl treatment is shown as **percentage to respective unstimulated osteoclasts** (unstimulated (100%) = continuous line). N (control/CIA rats) = osteoclast number: 10^-6^M (6/6), 10^-8^M (5/5), cathepsin K activity: 10^-6^M and 10^-8^M (6/6). Cells for osteoclastogenesis were seeded in triplicate and each supernatant was analyzed in duplicate. Box plots represent the 10^th^ to 90^th^ percentile of data sets. *p<0,05. CIA: collagen-induced arthritis, NKH 477: adenylyl cyclase activator, p.i.: post-immunization, TRAP: tartrate-resistant acid phosphatase

Fig 5 shows the results for NKH 477 treatment on osteoclasts generated from BMMs of CIA and control rats 40 days p.i. (results for day 20 are shown in Supporting Information File **S1 Fig**). Treatment with 10^-6^M NKH477 significantly suppressed formation of osteoclasts in cultures from CIA BMMs in relation to unstimulated cultures, whereas 10^-8^M NKH 477 by trend suppressed osteoclastogenesis in cultures from control and CIA BMMs (Fig 5A). In parallel, cathepsin K enzymatic activity was significantly reduced in osteoclast cultures from control and CIA rats treated with 10^-8^M NKH477 in relation to their respective unstimulated cultures, whereas 10^-6^M NKH 477 only inhibited cathepsin K activity in CIA osteoclasts (Fig 5B).

### 6. Matrix resorption assay and gene expression of activity markers

**Fig 6:**
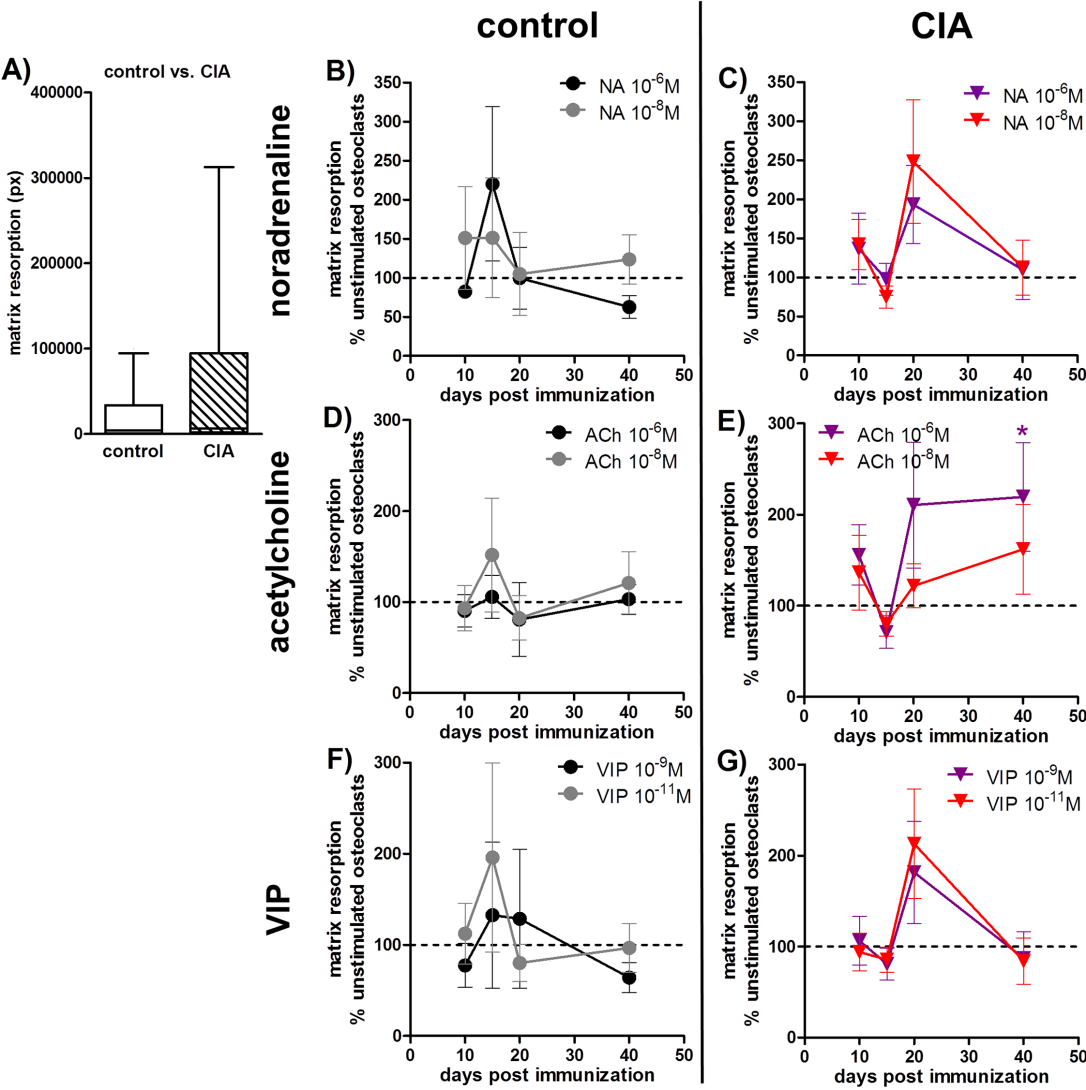
Matrix resorption assay. Osteoclasts were cultured on a bone-like matrix for 4 weeks and resorption was analyzed after von Kossa staining of remaining matrix. Matrix degradation of CIA osteoclasts 40 days p.i. and control osteoclasts 40 days post NaCl treatment is shown in **A.** N = 10 rats. **B-G) The effect of neurotransmitter stimulation on matrix degradative activity of osteoclasts from CIA and control rats is shown as percentage to respective unstimulated osteoclasts in the time-course of disease progression.** The results for NA are presented under **B** and **C,** for ACh under **D, E** and for VIP stimulation under **F, G** (unstimulated (100%) = dotted line). N (control/CIA rats) = NA 10^-6^M 10d (6/8) 15d (6/7) 20d (7/9) 40d (8/9); NA 10^-8^M 10d (6/8) 15d (6/8) 20d (7/9) 40d (8/9); ACh 10^-6^M 10d (6/8) 15d (6/8) 20d (7/8) 40d (7/9); ACh 10^-8^M 10d (6/7) 15d (6/8) 20d (7/8) 40d (8/9); VIP 10^-9^M 10d (6/8) 15d (6/8) 20d (7/9) 40d (8/9); VIP 10^-11^M 10d (5/7) 15d (6/7) 20d (7/9) 40d (8/9). Assay was performed in quadruplicate for each condition. Box plots represent the 10^th^ to 90^th^ percentile of data sets. Data points show mean ± SEM. *p<0,05 neurotransmitter vs. unstimulated; ^#^p<0,05 control vs CIA. ACh: acetylcholine, CIA: collagen-induced arthritis, NA: noradrenaline, p.i.: post-immunization, VIP: vasoactive intestinal peptide

Evaluation of active resorption of bone-like structures remains an indispensable tool for osteoclast-related studies. Osteoclasts generated from BMMs of rats with CIA showed no difference in matrix resorption activity in comparison to osteoclasts derived from BMMs of control animals which might be due to high inter-experimental variations (Fig 6A).

#### Neurotransmitter stimulation effects on matrix resorption activity are presented as percentage to the respective unstimulated cultures from CIA and control rats in the time-course of arthritis

10^-6^M ACh significantly enhanced matrix resorption of osteoclasts derived from CIA BMMs 40 days p.i. (Fig 6E) whereas stimulation with NA (Fig 6B and 6C) and VIP was without effect (Fig 6F and 6G).

However, osteoclasts from CIA rats had significantly lower mRNA levels of osteoclast activity markers like cathepsin K, matrix metalloproteinase (MMP)-9 and carbonic anhydrase II 10 and 15 days p.i. in relation to mRNA from osteoclasts derived from BMMs of NaCl-treated rats (Table 1 part D). Tartrate-resistant acid phosphatase (TRAP) was downregulated 15 and 20 days p.i. and the α3 subunit of the V-ATPase (*Tcirg1*) was affected 40 days p.i. (Table 1 part D).

## Discussion

Destruction of bone in rheumatic joints leading to constriction and disability is a major disease burden associated with RA. Mediators of this process are osteoclasts, the unique cell type which is able to degrade bone matrix. The main perspective of this study was to elucidate intrinsic changes in *in vitro*-osteoclastogenesis, differentiation and function of bone marrow-derived macrophages evoked by different stages of collagen-induced arthritis and the differential impact of sympathetic neurotransmitter stimulation.

Initially, to analyze if our *in vitro*-generated osteoclasts were equipped with suitable receptors in order to respond to neurotransmitter stimulation, we analyzed mRNA and protein expression of neurotransmitter receptors for ACh, NA and VIP. We detected a variety of neurotransmitter receptors expressed by osteoclasts and macrophages with mostly downregulated gene expression in CIA cells. Of note, existence of muscarinic receptors has not been reported for primary osteoclasts before, except for bone marrow derived macrophages [27], osteoblast-like cells and cell lines as well as in total bone tissue which were shown to express mRNA for various muscarinic ACh receptors [28]. Immunofluorescence staining of adrenoceptors, muscarinic ACh receptor M5 and PACAP receptor 1 revealed no differences in receptor location or intensity between osteoclasts from controls and CIA rats. However, that observation does not exclude alterations in downstream receptor activity which might affect cellular response to ligands.

We compared osteoclastogenesis, apoptosis and matrix resorptive- and cathepsin K activity of osteoclasts derived from BMMs of arthritic and healthy rats. Surprisingly, none of these readout parameters was affected by CIA although differentiation- and activity-associated genes were significantly down-regulated mostly before and at arthritis onset. Increased formation of osteoclasts was shown for cultures of peripheral blood mononuclear cells from RA patients associated with enhanced myelopoiesis [29] but Li and co-workers [30] demonstrated, using a human TNF-transgenic mice arthritis model, that enhanced osteoclast formation was confined to precursor cells isolated from blood and spleen and that osteoclastogenic potential of bone marrow precursors remained unaffected. They concluded that the higher mobilization of precursors from the bone marrow into the periphery is the causal effect for enhanced osteoclastogenesis in arthritis. This note is corroborated by our observations that native bone marrow cells from arthritic rats showed a significantly higher percentage of cells positive for macrophage markers CD11b and EMR1 in relation to bone marrow from healthy controls [27].

Kishimoto and coworkers found evidence that bone marrow cells isolated from rats with collagen-induced arthritis were less affected by disease mechanisms than synovial tissue as they found highly up-regulated pro-inflammatory cytokine mRNA as well as osteoclast markers cathepsin K, TRAP and Rank receptor which was much less apparent in bone marrow cells [31]. *In vivo* observations showed that patients with long-standing RA had elevated serum levels of cathepsin K [32], a protease which is released from activated osteoclasts into the acidified resorption lacuna where it degrades collagen fibrils. Inhibition of this catabolic enzyme protected mice with CIA from bone degradation [33]. However, it was not analyzed if higher serum cathepsin K levels were due to increased osteoclast numbers or increased production of this enzyme. In our system, cathepsin K activity was not altered in CIA osteoclast supernatants compared to osteoclasts from controls concordantly to unchanged osteoclastogenesis of CIA macrophages. Furthermore, observations from Akchurin et al. may explain the inconclusive results of our matrix degradation assay. They describe different and highly variable behavior of macrophage precursors and osteoclasts in long-term cultures (15–26 days) with different waves of osteoclast formation over time making it difficult to interpret results derived from these cultures [34].

When analyzing additional neurotransmitter effects on osteoclastogenesis, we observed enhanced osteoclast formation upon stimulation with ACh for osteoclasts derived from control and CIA BMMs. Tanaka et al. demonstrated a dual effect of nicotine on osteoclastogenesis [35]. In this study, exposure to nicotine enhanced osteoclast numbers and lead to the formation of a higher number of resorption pits. Because the effects were blocked by bungarotoxin, they were attributed to actions of the α7 nicotinic ACh receptor, which was not or only weakly detectable in our system (data not shown). Instead, we detected muscarinic receptor subtypes M3 and M5 which were not described in the context of osteoclastogenesis so far. Therefore, we assume that ACh actions might be rather mediated by these receptors or by other nicotinic receptor subtypes not included in our analysis. ACh mitogenic actions have been reported for rat astrocytes and human astrocytoma cells via muscarinic receptors [36]. The enhanced osteoclast formation upon ACh stimulation may therefore be partly a consequence of enhanced proliferation of osteoclast precursor cells. In a recent study we observed increased proliferation of BMM from healthy rats upon stimulation with ACh, whereas proliferation of BMMs from CIA rats was inhibited in the presence of ACh [27].

Of note, NA effects on osteoclast formation were dose-dependent: 10^-6^M NA, activating β-ARs, inhibited osteoclastogenesis of CIA and control BMMs, whereas 10^-8^M NA, activating α-ARs, enhanced osteoclast formation. Both concentrations showed a prominent timely variation in effectiveness: 10^-6^M/10^−8^ M NA affected CIA osteoclasts mainly at early disease time points whereas controls were affected at late and early time points post NaCl-treatment. So far in the literature, noradrenergic signaling via β-ARs was reported to enhance osteoclast formation in RAW264.7 and murine bone marrow cells via release of reactive oxygen species [9]; effects we would attribute to α-AR signaling in our system. Although Arai et al. showed that α-ARs 1B and 2B were expressed on human osteoclast-like multinucleated cells, they stated that treatment with the α1-agonist phenylephrine failed to induce any changes in osteoclastogenesis [10]. However, in their study the stimulatory pulse was set to terminally differentiated osteoclast-like cells, whereas we induced osteoclast differentiation in the presence of NA, what might explain the observed differences.

Another factor promoting longtime disease persistence in joints is the increased locally restricted expression of anti-apoptotic molecules like Bcl–2 and FLIP in synovial lining and pannus as well as down-regulation of pro-apoptotic Bim in synovial lining cells and resident macrophages [26, 37]. We observed upregulation of mRNA for pro-apoptotic c-myc 15 days p.i. and downregulation of anti-apoptotic Akt1 and Bcl–2. Analysis of caspase 3/7-mediated apoptosis revealed that osteoclasts from arthritic animals were only by trend more sensitive to caspase 3/7-mediated apoptosis at the acute disease time point. Additional neurotransmitter stimulation had little impact on apoptosis of CIA osteoclasts except for ACh which inhibited caspase 3/7 activity. In contrast, all three neurotransmitters, NA, ACh and VIP profoundly decreased caspase 3/7 activity in CIA BMMs being clearly cell protective for arthritic macrophages [27]. Contrary, caspase 3/7 activity in osteoclasts derived from control BMMs was clearly enhanced in the presence of both NA concentrations which is in line with our previous observations for control BMMs. These opposite effects of neurotransmitters in arthritic and control cells point to an altered reactivity to neurotransmitter stimulation which might be caused by changes in downstream receptor signaling pathways which are sensitive to an inflammatory environment but persist cell autonomous at least for several days after removal of the inflammatory stimulus.

Neurotransmitter stimulation had an impact on cathepsin K enzymatic activity. 10^-6^M ACh increased enzymatic activity in the cell culture supernatant of osteoclasts derived from control BMMs. VIP and NA instead decreased cathepsin K activity in CIA (NA and VIP) and control osteoclasts (VIP only). VIP has been described as a potent inhibitor of osteoclastogenesis acting rather on activity than on differentiation [38]. From our observations we can furthermore conclude, that osteoclasts derived from BMMs of CIA rats are more sensitive to neurotransmitters and thus react to lower concentrations of VIP. This note is corroborated by our observations that NA inhibitory effects on cathepsin K activity were only apparent in CIA osteoclasts with 10^-6^M being effective before arthritis onset until acute disease state and 10^-8^M affecting osteoclasts derived from acutely arthritic rats.

Evaluation of osteoclast numbers is not a conclusive indicator for their resorptive activity. Several studies on osteopetrotic mice, which display a high bone mass phenotype, show that osteoclast numbers are unchanged or even increased indicating that the development of this specific bone phenotype is associated with dysfunctional osteoclasts [39]. Noticeably, degradation of bone-like matrix of CIA osteoclasts was unaltered in relation to controls whereas in the presence of 10^-6^M ACh and 10^-8^M NA resorptive activity of CIA BMMs was significantly increased. However, gene expression of cathepsin K, TRAP, MMP–9, carbonic anhydrase II as well as V-ATPase subunit Tcirg1, all involved in catabolic activity of osteoclasts, was markedly reduced in osteoclasts generated from CIA animals. This discrepancy might be due to different time points for PCR sample collection (5 days after seeding) and resorption assay (4 weeks after seeding).

We suspected the cyclic AMP signaling pathway to be responsible for the inhibitory effects of VIP and NA on osteoclast cathepsin K activity and, for NA, dose-dependently on osteoclast formation, in analogy to inhibitory actions of calcitonin and forskolin via persistent activation of adenylyl cyclase [40, 41]. VIP and NA mainly signal through G_s_ protein-coupled receptor cascades activating adenylyl cyclase and increasing intracellular cAMP levels [15, 16]. Employing NKH477, a specific activator of adenylyl cyclase, significantly suppressed osteoclast formation and cathepsin K activity in osteoclasts derived from CIA and control rats. We would therefore suggest the regulation of cathepsin K activity mainly via cAMP-associated pathways whereas osteoclast differentiation presumably involves additional signaling pathways. ACh increases osteoclastogenesis and cathepsin K activity presumably via muscarinic ACh receptors M3 and M5 which is in line with our observations as these receptors are G_q_-coupled and lead to activation of phospholipase C and different downstream targets [42].

## Conclusions

Our data suggest that CIA does not affect metabolism and physiology of osteoclasts generated *in vitro* from BMMs isolated from arthritic rats. Although CIA clearly affects gene expression of osteoclasts in a time-dependent manner those alterations do not become evident on cellular level.

Contrary, we provide strong evidence that NA and VIP have mostly protective effects on bone matrix by inhibition of cathepsin K activity, an observation which is probably attributed to an increased cAMP signaling. Oppositely, we report an enhancing effect of ACh on cathepsin K and resorptive activity presumably mediated via muscarinic receptors. Expression of muscarinic receptors on osteoclasts and involvement in modulation of osteoclast cathepsin K activity has not been shown before. Increased osteoclast numbers upon ACh stimulation and a dose-dependent decrease in osteoclast numbers and increased caspase 3/7 reactivity by NA/VIP stimulation corroborates the assumption of opposite effects of the cholinergic and adrenergic nervous system on *in vitro* osteoclastogenesis and consequently CIA severity. We suggest that CIA modulates sensitivity towards neurotransmitter stimulation in osteoclasts enabling adaptation to locally changing neurotransmitter concentrations during disease pathogenesis.

## Material and Methods

### Animals

Female Dark Agouti rats were purchased from Janvier Labs (Le Genest St. Isle, France) at the age of 9 weeks. Rats were housed at 4 or 6 animals per cage and were allowed to adapt to animal laboratory conditions for one week. Rats were fed standard laboratory chow and water ad libitum and were kept under standard housing conditions in a 12h light-dark cycle. **All animal experiments were approved by and conducted according to institutional and governmental regulations for experimental animal usage (Ethical Review Committee, Government of the Oberpfalz Az. 54–2532.1-25/13).**

### Collagen-induced arthritis model

Prior to immunization, rats were anesthetized using the volatile narcotic isoflurane (Baxter, Unterschleißheim, Germany) followed by intramuscular application of a mixture of medetomidine (0.15mg/kg body weight (bw), Domitor, Orion Pharma Animal Health, Turku, Finland), midazolam (2mg/kg bw, Ratiopharm GmbH, Ulm, Germany) and fentanyl (5μg/kg bw, Rotexmedica, Trittau, Germany). For arthritis induction, 300μg dissolved bovine collagen type II (#804001-sol, MD Bioproducts, Egg, Switzerland) emulsified in an equal volume of incomplete Freund adjuvant (Sigma-Aldrich, Taufkirchen, Germany) was intracutaneously injected at the tail base. Controls received equal volume of 0.9% NaCl solution (Braun Melsungen AG, Melsungen, Germany). Anesthesia was antagonized by subcutaneous application of a mixture of atipamezole (0.75mg/kg bw, Antisedan, Orion Pharma Animal Health, Turku, Finland), flumazenil (0.2mg/kg bw, Hexal, Holzkirchen, Germany) and naloxone (0.12mg/kg bw, Ratiopharm GmbH, Ulm, Germany). Development of arthritis was monitored by determination of body weight and arthritis score at the respective sampling days (adapted from [43]).

### Isolation of bone marrow macrophages and *in vitro*-osteoclastogenesis

According to Muschter et al. [27], rats were killed using CO_2_; 10, 15, 20 and 40 days after immunization and control treatment and bone marrow was collected from femora and tibiae by centrifugation. Osteoclasts were generated using an adapted protocol kindly provided by Prof. Ulf Lerner (Umeå University, Sweden) [44]. Cells were centrifuged (5min, 245xg) and suspended in macrophage medium consisting of alpha-MEM with 10% fetal calf serum (FCS), 2% glutamate and 1% antibiotics/antimycotics (all Sigma-Aldrich, Taufkirchen, Germany) and 20ng/ml recombinant rat **M-CSF** (#400–28, Peprotech, Rocky Hill, NJ, USA). Cells were plated in a 100x20mm bacterial dish (#430591, Corning, Amsterdam, Netherlands) and cultivated at 37°C and 5% CO_2_. After 2 days, plates were washed with PBS and all non-adherent cells were removed. Attached cells, regarded as macrophages, were detached with 0.02% EDTA/PBS (Sigma-Aldrich, Taufkirchen, Germany) and 5min incubation on ice, followed by 1min at -20 degrees and a cell scraper. After centrifugation (245xg, 5min), cell number was determined with a Cedex automated cell counter (Roche Diagnostics GmbH, Mannheim, Germany). For respective experiments, macrophages were seeded at a density of 1.5x10^4^ cells/cm^2^. Osteoclastogenesis of BMM was induced with osteoclast medium consisting of macrophage medium additionally containing 50ng/ml recombinant rat **RankL** (#400–30, Peprotech, Rocky Hill, NJ, USA) for 5 consecutive days with one medium exchange after 3 days. Neurotransmitters (NT) were replenished simultaneously to medium exchange. Cultures in osteoclast medium without neurotransmitter supply are further referred to as unstimulated cultures. Acetylcholine chloride (**ACh**, #A22661), vasoactive intestinal peptide (**VIP**, #V6130) and L-(-)-norepinephrine (+)-bitartrate salt monohydrate (**NA**, #N5785) were purchased from Sigma-Aldrich (Taufkirchen, Germany).

### RNA isolation and quantitative PCR

Total RNA was isolated from osteoclasts after 5 days of differentiation using Stratagene’s Absolutly RNA Miniprep Kit (Stratagene, La Jolla, California, USA) according to the manufacturer’s instructions. For generation of cDNA, RNA was reversely transcribed using Affinity Script QPCR cDNA Synthesis Kit (Agilent Technologies, Santa Clara, CA, USA). PCR was performed with the Mx3005P QPCR System from Agilent Technologies using the Brilliant II SYBR Green QPCR Mastermix (Agilent Technologies, Santa Clara, CA, USA). Gene expression analysis was performed by relative quantification according to the ΔΔCT-method and normalization to GAPDH using RNA isolated from control osteoclasts as calibrator. The employed primers are listed in Supporting Information File **S1 Table**.

### Immunofluorescence staining of neurotransmitter receptors

10.000 macrophages per cavity of an 8 well-chamber slide (BD Biosciences, Heidelberg, Germany) were differentiated for 5 days in osteoclast medium. Subsequently, cells were fixed in 4% paraformaldehyde for 10min and washed with PBS. Until staining procedure, cells were stored at 4°C. Unspecific staining was blocked with 5% normal goat serum (NGS, Sigma-Aldrich, Taufkirchen, Germany) in PBS for 20min at RT. Neurotransmitter receptors were detected using: rabbit antibody against rat PACAP receptor 1 (1:50, sc–30018, Santa Cruz Biotechnologies, Dallas, TX, USA); rabbit antibody against muscarinic ACh receptor M5 (1:400, ab41171) and rabbit antibody against adrenoceptor β2 (3μg/ml, ab36956; both Abcam, Cambridge, UK), as well as rabbit antibodies against extracellular sections of adrenoceptors α1D (1:100, #AAR–019) and α2B (1:100, #AAR–021; Alomone Labs, Jerusalem, Israel). Antibodies were diluted in 1% NGS / PBS and incubated overnight at 4°C. Following washing, primary antibodies were incubated with F(ab’2) fragment of goat anti-rabbit IgG Alexa488-coupled (1:400, #A11070, Molecular probes/ Life Technologies, Eugene, OR, USA) diluted in 1% NGS / PBS for 1h at RT for visualization. Nuclei were counterstained with DAPI and cells were covered with Fluorescence Mounting Medium from Dako (Hamburg, Germany). Control staining using only the secondary antibody F(ab’)2-fragment revealed no unspecific immunofluorescence. Pre-blocking of α1D and α2B adrenoceptor antibodies with the respective blocking peptide, prevented staining and confirmed antibody specificity (data not shown). Specificity of antibodies against muscarinic ACh receptor M5, adrenoceptor β2 and PACAP receptor 1 were confirmed by western blotting (data not shown).

### Determination of osteoclast number

15.000 macrophages were seeded per cavity of a 24-well plate prepared with 12mm glass cover slips (Menzel-Gläser, Thermo Fisher Scientific, Braunschweig, Germany). Macrophages were cultured in osteoclast medium without neurotransmitter supply or with ACh, NA (final conc. 10^−6^ & 10^-8^M) or VIP (final conc. 10^−9^ & 10^-11^M) for 5 days. In studies involving the adenylyl cyclase activator NKH477 (cat.no. 1603, Tocris, Bristol, UK; final conc. of 10^−6^ and 10^-8^M), macrophages were differentiated for 5 days and additionally incubated in serum-free medium containing NKH477 for 24h (see section cathepsin K activity assay) and then further processed for determination of osteoclast number. Assay was performed in duplicate for each animal and condition, except for NKH477 experiments which were performed in triplicate. Afterwards, cells were fixed and stained for tartrate-resistant acid phosphatase (TRAP) using the “Acid phosphatase, Leukocyte (TRAP)” kit from Sigma-Aldrich (cat. No. 387A, Taufkirchen, Germany) following the manufacturer’s instructions. Cells containing three or more nuclei were defined as osteoclasts and were counted.

### Caspase 3/7 apoptosis assay

5000 macrophages/well were seeded in a black 96-well plate with clear bottom (BD Biosciences, Heidelberg, Germany) and differentiated in osteoclast medium for 5 days (without NT). Cells from each animal were analyzed in triplicate for each condition. Further procedure was performed according to a protocol from Muschter et al. [27].

### Cathepsin K activity assay

Enzymatic cathepsin K activity was assessed in cell culture supernatants using a substrate-based assay adapted from Wittrant et al. [45]. Briefly, 15.000 BMMs were differentiated in osteoclast medium without neurotransmitter supply or with the respective neurotransmitters (final conc. ACh, NA: 10^−6^ &10^-8^M, VIP: 10^−9^ & 10^-11^M) for 5 days. Studies involving the adenylyl cyclase activator NKH477 (cat.no. 1603, Tocris, Bristol, UK) were performed equally, using final concentrations of 10^−6^ and 10^-8^M NKH477. Assay was performed in duplicate for each animal and condition, except for NKH477 experiments which were performed in triplicate. Medium was exchanged for serum-free osteoclast medium with or without (w/o) neurotransmitters/NKH477. Supernatant was collected after 24h and either directly analyzed or stored at -80°C until use. Supernatant, reaction buffer (100mM sodium acetate, 1mM EDTA and 100nM DTT) and the non-fluorescent substrate Z-Leu-Arg-AMC (5μl per well / 500μM Stock, Peptanova, Sandhausen, Germany) were incubated in a 96-well plate at 37°C on a vertical shaker (150rpm) for 24h, protected from light. Each supernatant was analyzed in duplicate. Reaction was stopped (100mM Tris/100mM iodoacetic acid) and fluorescence was measured at 465nm (excitation 365nm). 7-amino-4-methyl coumarine (Sigma, Taufkirchen, Germany) served as standard. Protein content of the supernatant was determined using a bicinchoninic acid assay (BCA, Thermo Fisher, Waltham, MA, USA) according to the manufacturer’s instructions.

### Osteoclast resorption assay on bone-like matrix

Generation of a bone-like matrix was performed as described earlier [46]. Briefly, SAOS–2 cells were cultivated in 96-well plates in osteogenic medium consisting of αMEM with 10% heat inactivated FCS, 2% glutamate, 1% antibiotics/antimycotics, 300μM ascorbic acid 2-phosphate, 10mM β-glycerophosphate and 100nM dexamethasone. After 4 weeks, SAOS–2 cells were lysed by incubation in H_2_O_dest_ for at least 24h. To assess osteoclast resorption activity, plates were vigorously washed with H_2_O_dest_ to remove cell debris. 5,000 macrophages per cavity were cultivated in osteoclast medium w/o the respective neurotransmitters (final conc. ACh: 10^−6^ & 10^-8^M; NA 10^−6^ &10^-8^M, VIP 10^−9^ & 10^-11^M) for 4 weeks with 2 changes of medium / neurotransmitters per week. Assay was performed in quadruplicate for each condition and animal. For analysis, matrix was visualized by von Kossa staining.

### Von Kossa staining and analysis of resorption areas

Matrix plates were washed and cells were lysed in H_2_O_dest_. Vigorous washing removed cell debris and afterwards wells were incubated in 1% silver nitrate under UV light for a minimum of 3h. After washing, matrix was incubated in 5% sodium thiosulfate for 10min and again thoroughly washed. Matrix appeared black to dark brown. Whole wells were scanned with 100x magnification using the TissueFAXS system (DFG code: INST 89/341-1 FUGG) from TissueGnostics (Vienna, Austria). Pictures were blinded for the experimenter and resorption area was analyzed manually by encircling resorption pits using a touch pad (Bamboo Fun Pen & Touch, Wacom, Saitama, Japan) and Photoshop CS4 (Adobe, San José, CA, USA).

### Statistical analysis

Statistical analysis was performed using Prism 4 from GraphPad Software (San Diego, CA, USA). Time response data are displayed as mean ± SEM. Boxplots represent median (inner band of the box) and the tenth to ninetieth percentile (whiskers) of data sets. Non-parametric Wilcoxon signed rank test was used to test if median was significantly different to control conditions set to one hundred per cent. Variations between controls and CIA groups were compared using the two-tailed nonparametric Mann-Whitney test.

## Supporting Information

S1 Fig Influence of cAMP signaling on osteoclast number and cathepsin K activity. After 5 days of differentiation, osteoclasts were incubated in serum-free medium for another 24 hours and the collected supernatant was analyzed for cathepsin K enzyme activity. Remaining cells were fixed, stained for TRAP and cells containing ≥ 3 nuclei were counted as osteoclasts. The effect of adenylyl cyclase activator NKH 477 on osteoclasts number **(A)** and osteoclast cathepsin K enzymatic activity **(B)** from CIA rats 20 days p.i. and control rats 20 days post NaCl treatment is shown as percentage to respective non-stimulated osteoclasts (non-stimulated (100%) = continuous line). N (control/CIA rats) = osteoclast number: 10^-6^M (6/6), 10^-8^M (5/5), cathepsin K activity: 10^-6^M and 10^-8^M (6/6). Cells for osteoclastogenesis were seeded in triplicate and each supernatant was analyzed in duplicate. Box plots represent the 10^th^ to 90^th^ percentile of data sets. *p<0,05. CIA: collagen-induced arthritis, NKH 477: adenylyl cyclase activator, p.i.: post-immunization, TRAP: tartrate-resistant acid phosphatase
(TIF)

S1 Table Primer sequences for quantitative real-time PCR. (DOCX)

## Abbreviations

ACh: acetylcholine
BM: bone marrow
BMM: bone marrow-derived macrophage
cAMP: cyclic adenosine monophosphate
CIA: collagen-induced arthritis
mAChR: muscarinic ACh receptor
M-CSF: macrophage colony-stimulating factor
MMP–9: matrix metalloproteinase–9
NA: noradrenaline
NaCl: sodium chloride
NT: neurotransmitter
PACAP: pituitary adenylate cyclase-activating peptide
PCR: polymerase chain reaction
p.i.: post-immunization
RA: Rheumatoid Arthritis
RankL: receptor activator of NFκB ligand
RT: room temperature
SEM: standard error of the mean
TH: tyrosine hydroxylase
TRAP: tartrate-resistant acid phosphatase
VIP: vasoactive intestinal peptide
