# Supplementary figures and images for: Sympathetic Neurotransmitters Modulate Osteoclastogenesis and Osteoclast Activity in the Context of Collagen-Induced Arthritis
Source: PLoS One. 2015 Oct 2;10(10):e0139726. doi: 10.1371/journal.pone.0139726 (PMC4592252; doi:10.1371/journal.pone.0139726)

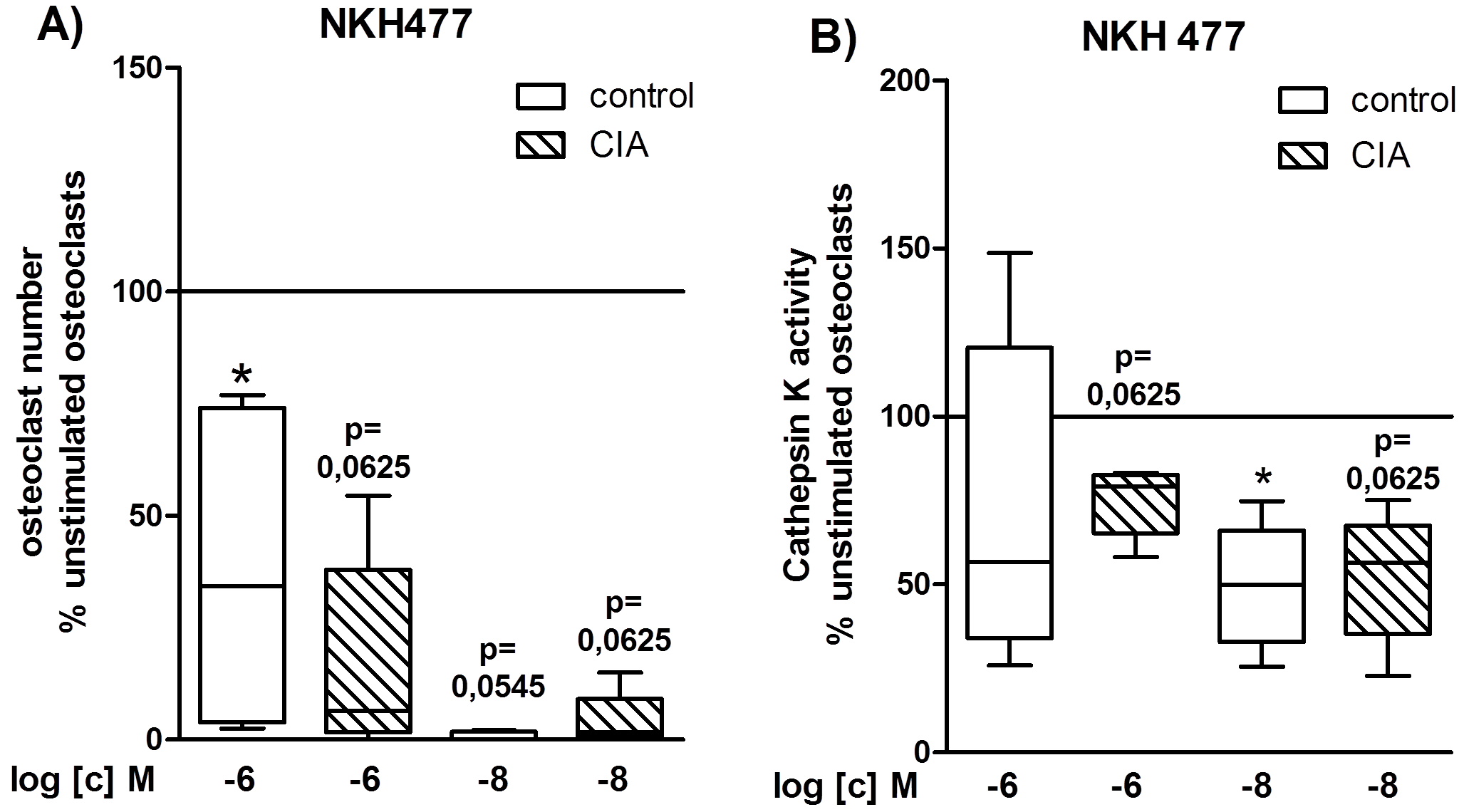

Supplement: S1 Fig — After 5 days of differentiation, osteoclasts were incubated in serum-free medium for another 24 hours and the collected supernatant was analyzed for cathepsin K enzyme activity. Remaining cells were fixed, stained for TRAP and cells containing ≥ 3 nuclei were counted as osteoclasts. The effect of adenylyl cyclase activator NKH 477 on osteoclasts number (A) and osteoclast cathepsin K enzymatic activity (B) from CIA rats 20 days p.i. and control rats 20 days post NaCl treatment is shown as percentage to respective non-stimulated osteoclasts (non-stimulated (100%) = continuous line). N (control/CIA rats) = osteoclast number: 10-6M (6/6), 10-8M (5/5), cathepsin K activity: 10-6M and 10-8M (6/6). Cells for osteoclastogenesis were seeded in triplicate and each supernatant was analyzed in duplicate. Box plots represent the 10th to 90th percentile of data sets. *p<0,05. CIA: collagen-induced arthritis, NKH 477: adenylyl cyclase activator, p.i.: post-immunization, TRAP: tartrate-resistant acid phosphatase (TIF) [file pone.0139726.s001.tif]
