# Supplementary material for: Sympathetic Neurotransmitters Modulate Osteoclastogenesis and Osteoclast Activity in the Context of Collagen-Induced Arthritis
Source: PLoS One. 2015 Oct 2;10(10):e0139726. doi: 10.1371/journal.pone.0139726 (PMC4592252; doi:10.1371/journal.pone.0139726)
Supplement: S1 Table — (DOCX) [file pone.0139726.s002.docx]

**Suppl. Table 1: Primer sequences used for quantitative real-time PCR**

| **Gene name** | **Primer sequence [5‘🡪3‘]** | **Tm [°C]** | **Amplicon length [bp]** | **Acc.No.** |
| --- | --- | --- | --- | --- |
| **Osteoclast differentiation and activity markers** | | | | |
| Cathepsin K | Fwd: AGACGCTTACCCGTATGTGG | 59.4 | 151bp | NM_031560 |
|  | Rev: GGACACAGAGACGGGTCCTA | 61.4 |  |  |
| Calcitonin receptor | Fwd: GATGCTGTGTGAGGGGATCT | 59.4 | 152 bp | NM_001034015 |
|  | Rev: CGTTGTAGTAGACGGCACGA | 59.4 |  |  |
| Carbonic anhydrase II | Fwd: TGGTTCACTGGAACACCAAA | 55.3 | 100 bp | NM_019291 |
|  | Rev: GAGGCAGGTCCAATCTTCAA | 57.3 |  |  |
| MMP-9 | Fwd: TTCGACGCTGACAAGAAGTG | 61.4 | 156 bp | NM_031055 |
|  | Rev: AGGGGAGTCCTCGTGGTAGT | 57.3 |  |  |
| NFATc1 | Fwd: ACGATGTGGAGGTGGAAGAC | 59.4 | 161 BP | XM_002725364 |
|  | Rev: CTCATAGGAGGACGCCTCAG | 61.4 |  |  |
| tartrate-resistent acid phosphatase (TRAP) | Fwd: GAGAACGGTGTGGGCTATGT | 59.4 | 131 bp | NM_019144 |
|  | Rev: GTGAAGCCACCCAGAGAGTC | 61.4 |  |  |
| ATPase proton pump subunit (Tcirg1) | Fwd: CCTCCTACTTGCGTCTCTGG | 61,4 | 261 bp | NM_199089.2 |
|  | Rev: TGTAGCCGGTCCCTGAATAG | 59,4 |  |  |
| Rank receptor | Fwd: AGGGAAAACGCTGACAGCTA | 59,3 | 171 bp | NM_001271235 |
|  | Rev: ACCATCTTCTCCTCCCGAGT | 59,7 |  |  |
| M-CSF receptor (csfr) | Fwd: TACTGCTGCTACGGAGACCT | 57,5 | 147 bp | NM_001029901.1 |
|  | Rev: ACTGTCCCTGCGCACATATT | 57,5 |  |  |
| **Neurotransmitter receptors** | | | | |
| VIP receptor 1 | Fwd: ATCCTCCTCTCCATCCTGGT | 59,1 | 140 bp | NM_012685.2 |
|  | Rev: ATGAGCAGAAGCGTGGACTT | 59,7 |  |  |
| VIP receptor 2 | Fwd: CAGATGTTGGTGGCAATGAC | 57,4 | 165 bp | NM_017238 |
|  | Rev: CCTGGAAGGAACCAACACAT | 57,7 |  |  |
| PACAP receptor 1 | Fwd: ACGTCAGCAAGAGGGAAAGA | 59,0 | 136 bp | NM_133511 |
|  | Rev: ACCTTCCAGCTCCTCCATTT | 58,6 |  |  |
| M3 muscarinic AChR | Fwd: ACAGCTGCATACCCAAAACC | 58,7 | 205 bp | NM_012527 |
|  | Rev: GGCACTCGCTTGTGAAAAAT | 57,6 |  |  |
| M5 muscarinic AChR | Fwd: CAG AGA AGC GAA CCA AGG AC | 59,4 | 236 bp | NM_017362.4 |
|  | Rev: CTC AGC CTT TTC CCA GTC AG | 59,4 |  |  |
| adrenoceptor α1D | Fwd: CAA CCT GCT GGT CAT CCT TT | 58,1 | 233 bp | NM_024483.1 |
|  | Rev: AGATGGTGCAGAGGCTAAGG | 59,2 |  |  |
| adrenoceptor α2A | Fwd: GTGTGTTGGTTCCCGTTCTT | 58,6 | 149 bp | NM_012739 |
|  | Rev: CGGAAGTCGTGGTTGAAAAT | 56,7 |  |  |
| adrenoceptor α2B | Fwd: AGTTTTCGTGGTCTGCTGGT | 58,8 | 163 bp | NM_138505.2 |
|  | Rev: GTCCTGGTTGAAGACGGTGT | 59,9 |  |  |
| adrenoceptor β2 | Fwd: CACATCGCCCTTCAAGTACC | 59,6 | 116 bp | NM_012492 |
|  | Rev: ACCAGTGCATCTGGATAGGC | 59,5 |  |  |
| **Apoptosis/survival-related signaling pathways** | | | | |
| Bcl2 | Fwd: GGGAGCGTCAACAGGGAGATG | 63.7 | 169 bp | NM_016993.1 |
|  | Rev: GACAGCCAGGAGAAATCAAACAGAG | 63.0 |  |  |
| Akt1 | Fwd: CACCGTGTGACCATGAACGA | 57,5 | 238 bp | NM_033230.2 |
|  | Rev: GGTCGTGGGTCTGGAATGAG | 59,5 |  |  |
| Mapk1 (Erk1) | Fwd: TGCTTTCTCTCCCGCACAAA | 55,4 | 185 bp | NM_053842.1 |
|  | Rev: AGCAATGGGCTCATCACTTG | 55,4 |  |  |
| Mapk3 (Erk2) | Fwd: AACTCCAAGGGCTACACCAA | 55,4 | 285 bp | NM_017347.2 |
|  | Rev: GGTTAACATCCGGTCCAGCA | 57,5 |  |  |
| c-Myc | Fwd: TCGCCCAAATCCTGTACCTC | 57,5 | 160 bp | NM_012603.2 |
|  | Rev: CATCTTGTTCTTCTTCAGAGTCGC | 57,9 |  |  |
| **Housekeeping Gene** | | | | |
| GAPDH | Fwd: TTG GTG GTG CAG GAT GCA TTG C | 61,2 | 291 bp | NM_017008.3 |
|  | Rev: CAC AGT CAA GGC TGA GAA TGG G | 64,5 |  |  |
